# Off‐the‐Shelf Synthetic Biodegradable Grafts Transform In Situ into a Living Arteriovenous Fistula in a Large Animal Model

**DOI:** 10.1002/adhm.202303888

**Published:** 2024-03-26

**Authors:** Paul J. Besseling, Wojciech Szymczyk, Martin Teraa, Raechel J. Toorop, Dan Jing Wu, Rob C. H. Driessen, Arturo M. Lichauco, Henk M. Janssen, Melanie van de Kaa, Krista den Ouden, Petra M. de Bree, Joost O. Fledderus, Carlijn V. C. Bouten, Gert J. de Borst, Patricia Y. W. Dankers, Marianne C. Verhaar

**Affiliations:** ^1^ Department of Nephrology and Hypertension Regenerative Medicine Center Utrecht University Medical Center Utrecht Utrecht University Utrecht 3584 CX the Netherlands; ^2^ Department of Vascular Surgery University Medical Center Utrecht Utrecht 3584 CX the Netherlands; ^3^ Department of Biomedical Engineering and Institute for Complex Molecular Systems Eindhoven University of Technology Eindhoven 5612 AZ the Netherlands; ^4^ Mechanobiology Services Eindhoven Department of Biomedical Engineering Eindhoven University of Technology Eindhoven 5612 AZ the Netherlands; ^5^ SyMO‐Chem B.V Eindhoven 5612 AZ the Netherlands

**Keywords:** AV shunt, biodegradable supramolecular material, biofunctionalization, goat model, in situ tissue engineering, vascular dialysis access

## Abstract

Current vascular access options require frequent interventions. In situ tissue engineering (TE) may overcome these limitations by combining the initial success of synthetic grafts with long‐term advantages of autologous vessels by using biodegradable grafts that transform into autologous vascular tissue at the site of implantation. Scaffolds (6 mm‐Ø) made of supramolecular polycarbonate‐bisurea (PC‐BU), with a polycaprolactone (PCL) anti‐kinking‐coil, are implanted between the carotid artery and jugular vein in goats. A subset is bio‐functionalized using bisurea‐modified‐Stromal cell‐derived factor‐1α (SDF1α) derived peptides and ePTFE grafts as controls. Grafts are explanted after 1 and 3 months, and evaluated for material degradation, tissue formation, compliance, and patency. At 3 months, the scaffold is resorbed and replaced by vascular neo‐tissue, including elastin, contractile markers, and endothelial lining. No dilations, ruptures, or aneurysms are observed and grafts are successfully cannulated at termination. SDF‐1α‐peptide‐biofunctionalization does not influence outcomes. Patency is lower in TE grafts (50%) compared to controls (100% patency), predominantly caused by intimal hyperplasia. Rapid remodeling of a synthetic, biodegradable vascular scaffold into a living, compliant arteriovenous fistula is demonstrated in a large animal model. Despite lower patency compared to ePTFE, transformation into autologous and compliant living tissue with self‐healing capacity may have long‐term advantages.

## Graphical abstract

In situ tissue engineering combines the initial success of synthetic grafts with the long‐term advantages of autologous vessels by using biodegradable grafts that transform into autologous vascular tissue at the site of implantation. After 3 months, these synthetic, biodegradable vascular scaffolds are remodeled into a living, compliant arteriovenous fistula in a large animal model.

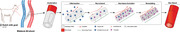

## 1 Introduction

Vascular access is considered the Achilles’ heel of hemodialysis due to a significant risk of loss of patency of the access conduit, which often requires recurring interventions.^[^ ^1^ ^]^ Autologous arteriovenous (AV) fistulas are generally the preferred vascular access option. However, their use is limited by pre‐existing disease, previous harvesting, unsuitability of the vessels, and/or maturation failure.^[^ ^2^ ^]^ Although current synthetic alternatives like expanded polytetrafluoroethylene (ePTFE) are off‐the‐shelf available and immediately useable, they are non‐compliant and susceptible to infection, intimal hyperplasia, and/or thrombosis.^[^ ^3^, ^4^, ^5^ ^]^

Vascular tissue engineering (TE) may provide a promising alternative.^[^ ^6^, ^7^ ^]^ Previous studies on vascular access TE have shown compelling results by growing vascular grafts in a bioreactor in vitro^[^ ^8^, ^9^, ^10^ ^]^ or by exploiting the foreign body response to create tubular fibrous tissues in vivo, which are subsequently implanted as an AV graft.^[^ ^11^, ^12^ ^]^ However, both in vitro and in vivo approaches use lengthy, multistep creation processes with cell harvesting and/or maturation phases. An in situ approach is a potentially promising alternative in which the body itself functions as the bioreactor.^[^ ^13^, ^14^ ^]^ In situ vascular TE uses synthetic, porous, biodegradable vascular scaffolds, which in vivo transform into living vascular tissue.^[^ ^13^, ^15^, ^16^ ^]^ The success of this remodeling process relies on maintaining a balance between the degradation of the scaffold and neo‐tissue formation.^[^ ^17^ ^]^ Rapid degradation may result in deformations,^[^ ^18^ ^]^ whereas long‐term scaffold presence can result in aberrant tissue formation like fibrosis.^[^ ^19^, ^20^ ^]^ To counteract this adverse remodeling, several studies have explored pre‐seeding scaffolds with cells; such as bone marrow^[^ ^21^ ^]^ or mononuclear cells.^[^ ^15^, ^17^ ^]^ Alternatively, intelligent scaffold materials can be applied that can be modified and functionalized to facilitate the transformation from synthetic vascular tissue while maintaining the balance between scaffold breakdown and neo‐tissue formation.

We adopted a cell‐free in situ TE approach using a micro fibrous electrospun vascular scaffold composed of a modular supramolecular material. To prevent kinking of the graft we implemented a biodegradable coil design made by 3D printing PCL. This has been previously shown by Wu et al., to be the optimal anti‐kinking design for lumen stability, with a minimum bending radius of 4 mm.^[^ ^22^ ^]^

The supramolecular elastomeric material is composed of polycarbonate (PC) chain‐extended with bisurea (BU) units. Previously we have successfully applied these BU materials in in situ tissue engineering approaches of vascular grafts in small animals^[^ ^23^, ^24^ ^]^ and of heart valves in sheep.^[^ ^25^ ^]^ Importantly, the presence of BU units in the main chain allows for a modular approach in which additives functionalized with BU moieties can be mixed‐and‐matched so that the mechanical properties can be tuned,^[^ ^26^ ^]^ as well as functionalization with for example bioactive^[^ ^27^ ^]^ or anti‐fouling additives^[^ ^28^ ^]^ can be performed. These highly adjustable characteristics could help address the challenges of infection, intimal hyperplasia, remodelling, and/or thrombosis that are associated with fully synthetic grafts. We previously demonstrated that non‐covalent functionalization of scaffold material with short peptides based on stromal cell‐derived factor‐1α‐based (SDF1α), a chemoattractant of lymphocytes, monocytes, and progenitors’ cells, enhanced the cellularization of implanted small diameter grafts in a rat model.^[^ ^29^, ^30^ ^]^

Here, we investigated in a large animal model, the ability of a fully biodegradable, cell‐free, synthetic biodegradable AV‐graft with or without SDF1 biofunctionalization, to remodel within 3 months into an autologous, living, neo‐vessel with self‐healing capacity and compliance.

## 2 Results

### 2.1 Scaffold Design

**Figure 1:**
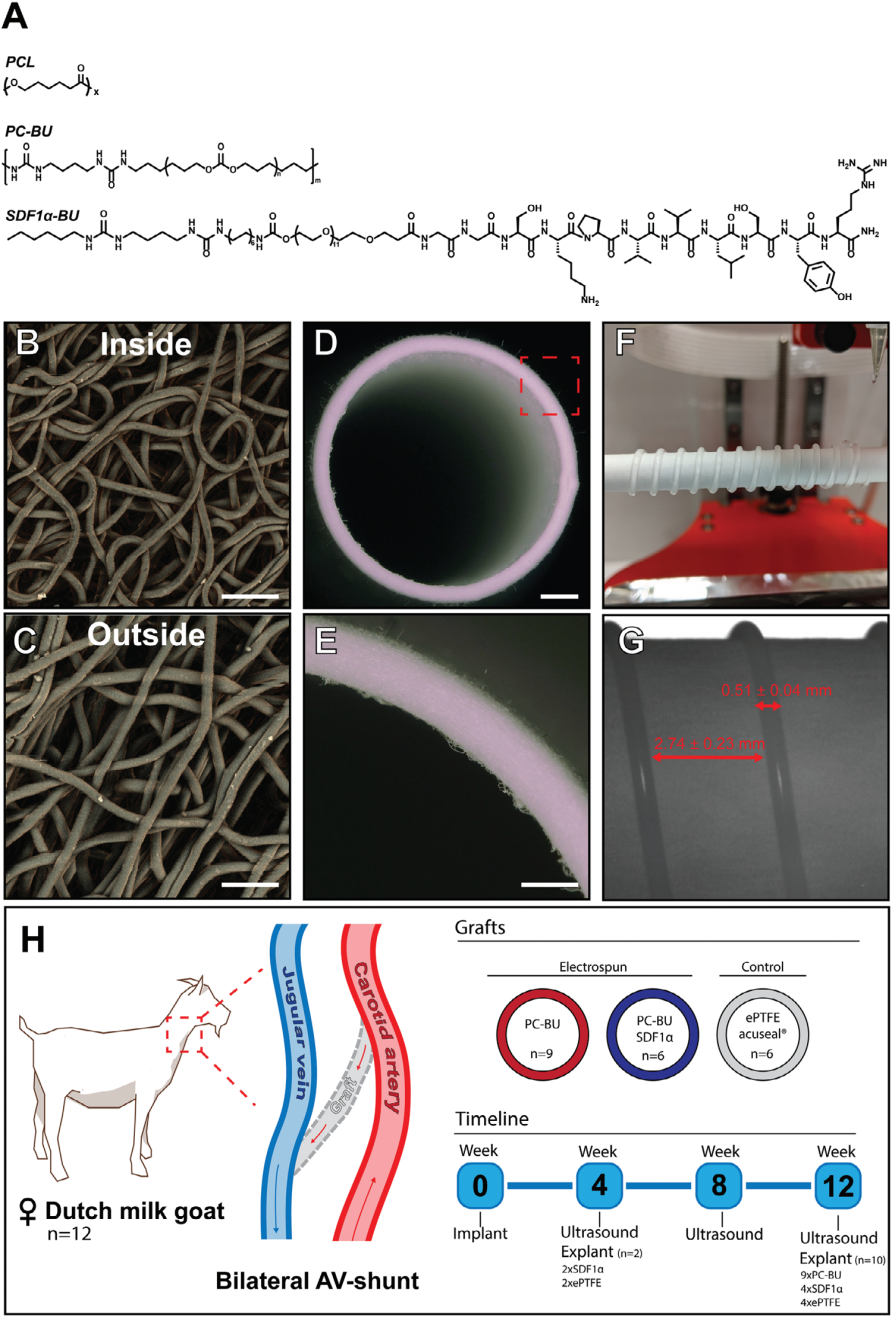
Experimental set‐up and graft creation. A) Chemical structures of PCL (M_n_ = 45 kg mol^−1^), PC‐BU (M_n_ = 21 kg mol^−1^), and a BU modified SDF1α derived peptide. Electrospun fibers (3.5 µm) in random configuration on the B) inside and C) outside of D) the graft are spun into a tubular structure, E) with an average 500 µm wall thickness. A reinforcing spiral, made of 3d printed PCL, is sandwiched between the electrospun layers to prevent kinking. F) Spacing 2.75 mm and a thickness of G) 0.50 mm. Grafts (made of PC‐BU, with (*n* = 9) or without SDF1α peptide biofunctionalization (*n* = 6), and ePTFE controls (*n* = 6) are implanted bilateral as an arteriovenous graft in 12 female Dutch milk goats in a straight configuration between the Carotid artery and Jugular vein. H) Grafts are explanted at 4‐ and 12‐week endpoints and imaged every 4 weeks by ultrasound. Scale bars represent 25 µm (B, C), 1 mm (D), and 500 µm (E).

The luminal layer was made of electrospun PC‐BU (± BU‐SDF1α) (PC‐BU: M_n_ = 21 kg mol^−1^) (**Figure** **1**A) with a thickness of 450 ± 50 µm (Figure 1E). Directly onto this luminal electrospun layer, an anti‐kinking spiral made of PCL (PCL: M_n_ = 45 kg mol^−1^) (Figure 1A) was 3D printed (Figure 1F), with a thickness of 0.51 ± 0.04 mm and an interspacing of 2.74 ± 0.23 mm (Figure 1G). On top of that, the adventitial layer, with a thickness of 102 ± 20 µm, made of electrospun PC‐BU(±SDF1α) was applied, sandwiching the 3D printed spiral between the luminal and adventitial electrospun layer. The fiber diameter at the luminal side of the bare PC‐BU and PC‐BU‐SDF1α grafts was determined to be 3.7 ± 0.6 and 3.7 ± 0.5 µm, respectively (Figure 1B). The fiber diameter of the adventitial layer of the PC‐BU and PC‐BU‐SDF1α grafts were observed to be 3.9 ± 0.4 µm (Figure 1C) and 3.8 ± 0.6 µm respectively. The lumen diameter of the grafts was 5.7 ± 0.2 mm, independently of the incorporation of stromal cell‐derived factor‐1α (SDF1α).

### 2.2 Animal Survival and General Graft Characteristics

**Figure 2:**
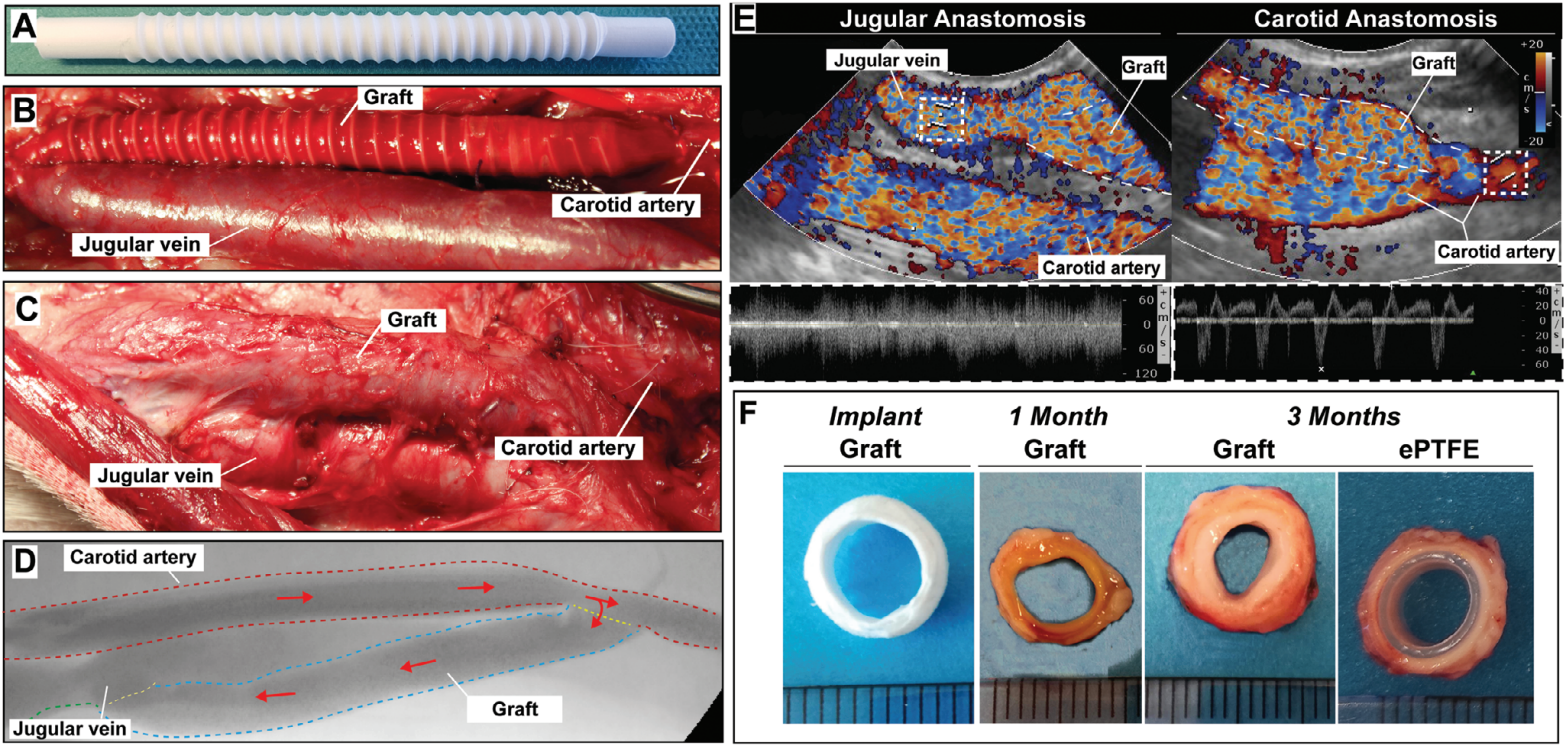
Graft implantation, visualization and explantation. A,B) Synthetic grafts are implanted between the carotid artery and jugular vein end‐to‐side in a straight configuration. C) After 1 and 3 months flow is visualized by D) angioCT and E) color Doppler and the grafts are explanted. F) Cross sections of the grafts at implantation and explantation show the rate of tissue formation and remodeling (scale 1 mm).

**Figure 3:**
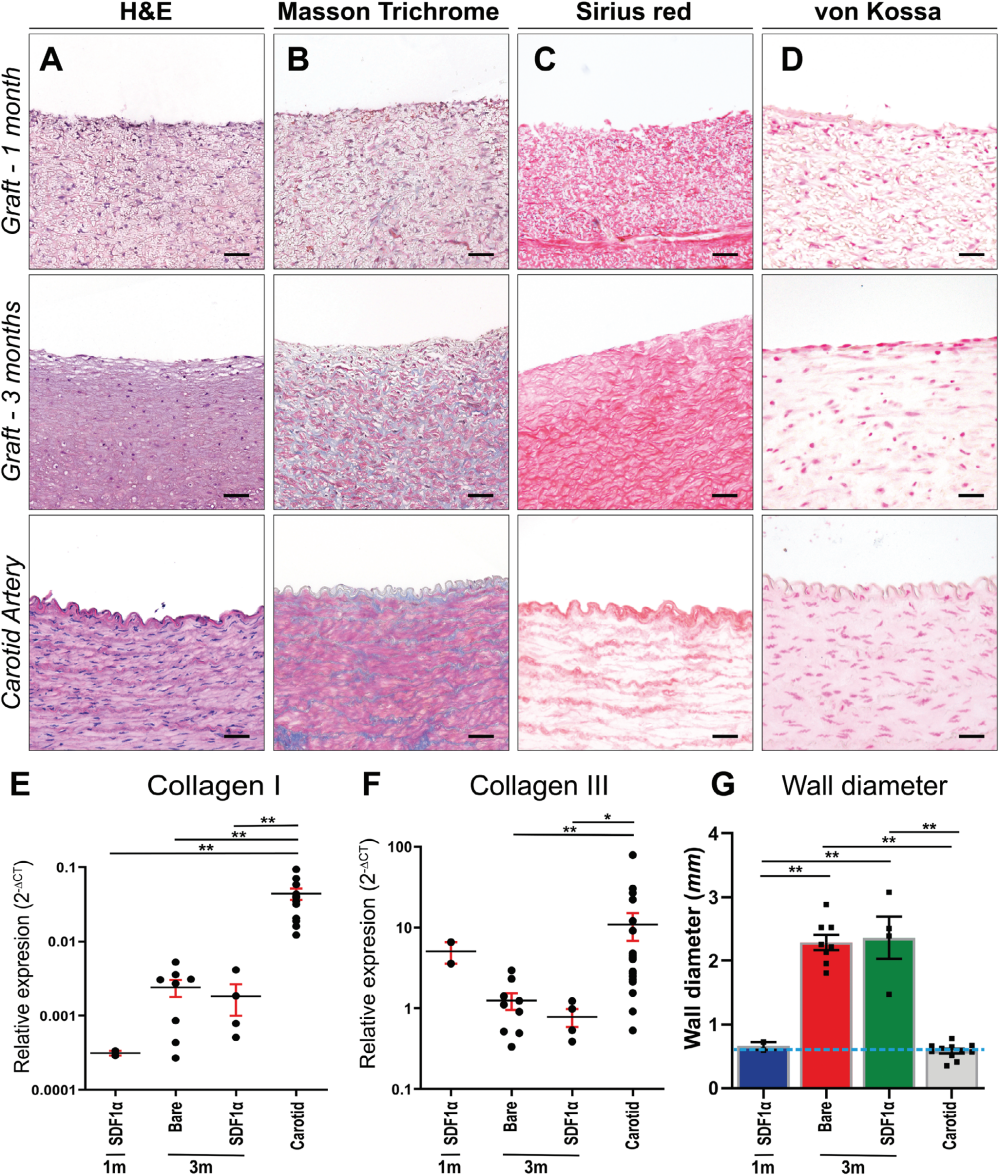
Histological evaluation on representative transverse sections of the center part of the grafts, explanted at 1 and 3 months and the native carotid artery as control. A) Stained with H&E, B) Masson's Trichrome Verhoef (cytoplasm (pink), collagen (blue), and nuclei (dark blue)). C,D) Picro sirius red to visualize collagen and von Kossa and nuclear fast red to visualize calcifications (black). Relative gene expression (2Δ‐CT) of E) Collagen I and F) Collagen III compared between (SDF1α peptide) grafts at 1 month (*n* = 2), 3 months (Bare *n* = 9, SDF1α *n* = 4), and native carotids (*n* = 12) as controls. G) Wall diameter in mm, with wall diameter of graft implant (0.6 mm) represented by dashed line. Electrospun fibers are visible in white. Scale bars 50 µm. Data are shown as mean ± SEM. *p < 0.05, **p < 0.01.

Surgical handling and suturing of the grafts were considered easy by our vascular surgeons. There was little to no bleeding from the anastomosis regions, and oozing through the porous structure of the graft wall could be resolved by light gauze pressure or absorbable surgicel. Grafts were explanted in 10 animals at 3 months (Bare *n* = 9; SDF1α *n* = 4; ePTFE *n* = 4) and 2 animals at 1 month (SDF1α *n* = 2; ePTFE *n* = 2) due to early termination as a result of suspected (aspiration) pneumonia. No deformations (aneurysm, dilation, and/or ruptures) were observed at explantation and explants showed a clear remodeling from synthetic to neo‐tissue (**Figure** **2**F). Wall thickness was significantly increased at 3 months (Bare, *n* = 8, 2.29 ± 0.12 mm—SDF1α, *n* = 4 2.36 ± 0.33 mm vs 0.61 ± 0.50 mm before implantation, *p* < 0.01) (**Figure** **3**G).

### 2.3 Extracellular Matrix Formation in the Grafts

Hematoxylin and Eosin (H&E) staining (Figure 3A) showed an even distribution of cells throughout the graft at both 1 month and 3 months, with sparse proliferating (KI67^+^) cells (Figure S2, Supporting Information). Only in the sections of grafts explanted after 1 month electrospun fibers (white dots) were still clearly visible. Masson's Trichrome staining (Figure 3B) indicated an increase in connective tissue deposited in the graft over time, which is in line with the high collagen presence observed with the SR staining (Figure 3C). The Von Kossa staining (Figure 3D) showed no calcifications in the grafts or native carotid tissue. Extracellular matrix (ECM) marker Collagen III, associated with early‐stage vascular *TE*, gene expression levels in grafts were lower (*p* < 0.05) compared to native carotid tissue. Expression of Collagen I, a collagen type associated with later stages of neo‐tissue formation showed an increase over time toward native levels (*p* < 0.01) (Figure 3F).

### 2.4 Vascular Markers

**Figure 4:**
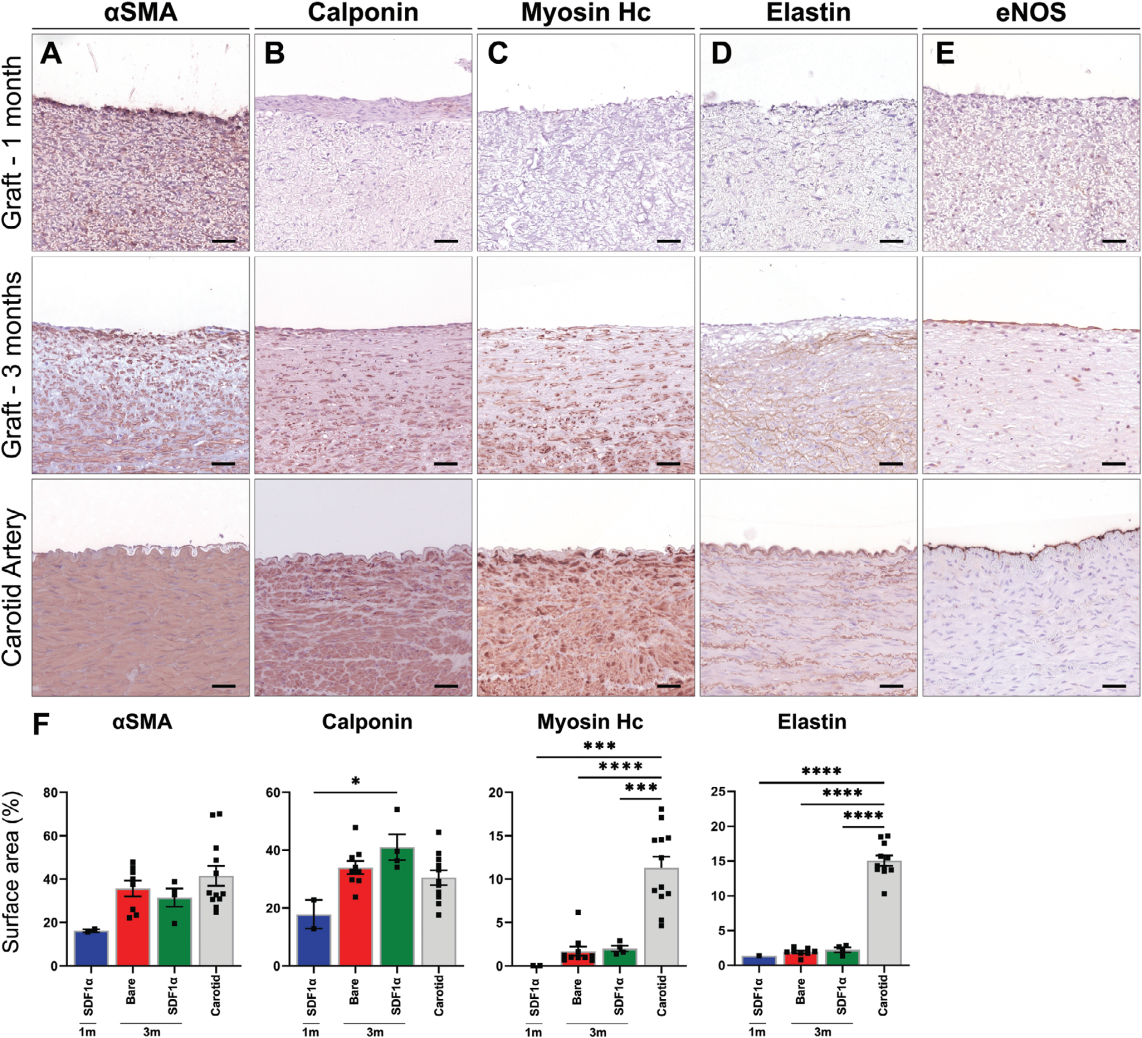
Immunohistochemistry on vascular markers in representative transverse sections of the center part of the grafts, at 1 month (*n* = 2), 3 months (Bare *n* = 9, SDF1α *n* = 4), and native carotids (*n* = 12) as controls. Stained with Novared for A) smooth muscle marker αSMA, B) early contractile marker Calponin, C) mature contractile marker Myosin Heavy Chain, D) Elastin and E) endothelial marker eNOS. With measured positive surface area of whole cross sections taken of separate parts of the grafts. Electrospun fibers are visible in white. Scale bars 50 µm. Data are shown as mean ± SEM. *p < 0.05, ***p < 0.001, ****p < 0.0001.

At 1 and 3 months, grafts contained smooth muscle marker alfa smooth muscle actin (αSMA) (**Figure** **4**A) positive cells, similar to the native carotid artery. The presence of cells positive for the intermediate contractile marker calponin (Figure 4B) significantly increased from 1 to 3 months (1 m SDF1α, *n* = 2, 16.3 ± 3.4%—3 m Bare, *n* = 9, 34 ± 2.3%—3 m SDF1α, *n* = 4, 41.0 ± 4.5%). At 3 months mature contractile markers, i.e., Myosin Heavy Chain (MYH) (Bare; 1.7 ± 0.5%, SDF1α; 1.99 ± 0.34%) (Figure 4C) and Elastin (Bare; 1.9 ± 0.2%, SDF1α; 2.2 ± 0.3%) (Figure 4D), were present, although significantly (*p* < 0.01) lower compared to native carotid artery tissue (*n* = 12, MYH:11.8 ± 1.3%, Elastin: 15.1 ± 0.8%). In all graft sections, a layer of endothelial cells at the luminal site was observed as shown by eNOS staining. Furthermore, endothelial lining and capillary ingrowth were evident from the presence of eNOS and VWF positive cells in the medial layer of the grafts (Figure 4E and Figure S3, Supporting Information).

### 2.5 Immune Cell Presence in the Grafts

**Figure 5:**
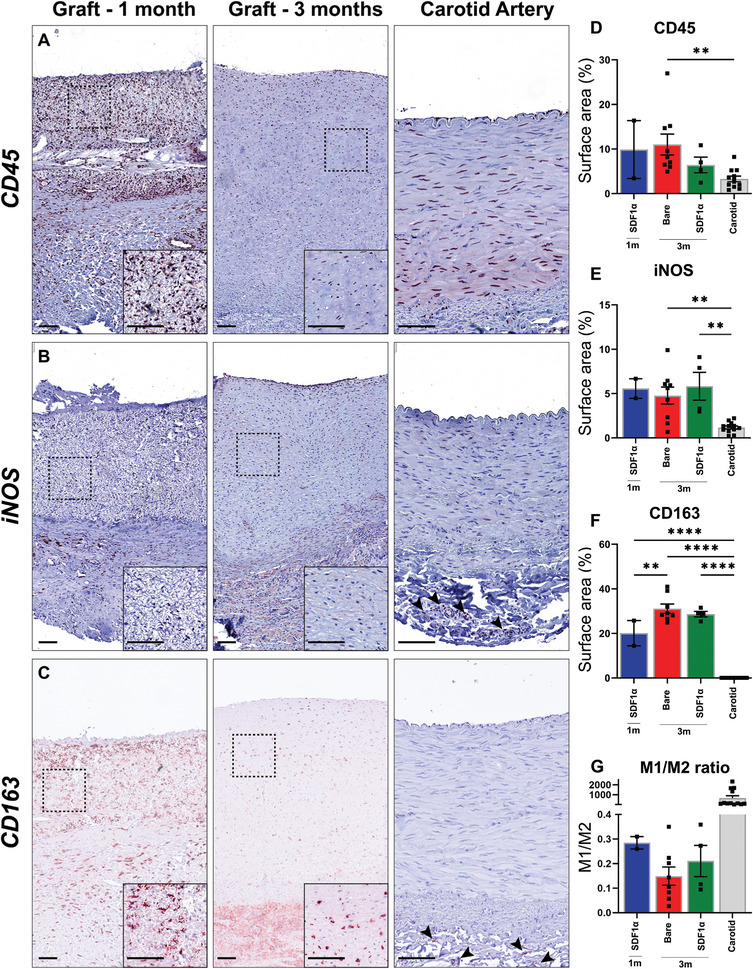
Macrophage infiltration and immune markers. A) Immunohistochemistry on leukocytes (CD45^+^), B) M1 (inflammatory) macrophages (iNOS^+^), and C) M2 (anti‐inflammatory) macrophages (CD163^+^) with Novared in representative transverse sections of the center part of the grafts explanted at 1 (*n* = 2) and 3 months (Bare *n* = 9, SDF1α *n* = 4), and native carotids (*n* = 12). Positive surface area of whole cross sections of the grafts for D) CD45^+^, E) iNOS^+^, and F) CD163^+^. M1/M2 ratio as an indicator of inflammation versus neo‐tissue formation marker (CD68^+^), compared between (SDF1α peptide) grafts at 1 month and 3 months and native carotids as controls (C). Electrospun fibers are visible in white. Scale bars 100 µm. Data are shown as mean ± SEM. **p < 0.01, ***p < 0.001, ****p < 0.0001.

Markers of the inflammatory response, including CD45 for leukocytes, iNOS for M1 macrophages, and CD163 for M2 macrophages were abundantly present within the formed neo‐tissue in both 1‐ and 3‐month explants. Histological evaluation demonstrated a higher surface positivity of leukocyte marker CD45^+^ in graft sections (1 m, *n* = 2, 9.8 ± 6.48%; 3 months bare, *n* = 9, 11 ± 2.34%; SDF1α, *n* = 4, 6.40 ± 1.76), compared to the native carotid (*n* = 12, carotid 3.29 ± 0.62%), with the bare graft at 3 months showing significantly higher levels (*p*: < 0.01). The presence of both inflammatory M1 (iNOS^+^) and M2 (CD163^+^) macrophages were significantly (*p*: < 0.01) higher compared to the native carotid artery. The surface area of iNOS^+^ cells did not differ over time (1 m 5.57 ± 1.11%; 3 months bare 4.77 ± 0.96%) or was influenced by SDF1α biofunctionalization (3 months SDF1α 5.82 ± 1.57%). The surface area of CD163^+^ positive macrophages, associated with neo‐tissue formation and proliferation, in the explanted grafts, increased over time (1 month; 20.1 ± 5.6% vs 3 months Bare: 31.1 ± 2.1% & SDF1α: 28.6 ± 5.7%) (**Figure** **5**A,B), but did not differ between bare and SDF1α grafts. The M1/M2 ratio (1 month: 0.28 ± 0.03, 3 months bare: 0.15 ± 0.04, 3 months SDF1α: 0.21 ± 0.06) indicated a clear favor in the presence of tissue building CD163^+^ cells compared to cells positive for inflammatory M1 marker iNOS in explanted grafts sections. Both iNOS^+^ and CD163^+^ cells in explanted graft sections at 3 months were predominantly observed in the media and showed low presence in the newly formed neo‐intima. In native carotid sections iNOS^+^ and CD163^+^ cells were scarce and were predominately found in the adventitial layer.

### 2.6 Mechanical Properties and Morphology

**Figure 6:**
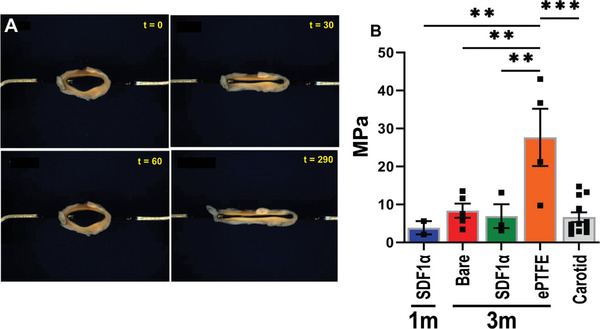
Mechanical characterization by uniaxial ring test. A) Example images of ring test during stretching by ring test of a graft explanted after 3 months. B) Estimated modulus (MPa) calculated form ring test displacement of center part of the grafts explanted at 1 (*n* = 2) and 3 months (Bare *n* = 5, SDF1α *n* = 3), ePTFE (*n* = 4) and native carotids (*n* = 12). **p < 0.01, **p.

Mechanical properties of implanted and explanted grafts were assessed by uniaxial ring testing (**Figure** **6**A). During multiple stretches, grafts returned to their original morphology (Video S1, Supporting Information) and showed an estimated modulus of 3.85 ± 1.75 MPa at 1 month (*n* = 2) and proceeded to 6.90 ± 3.13 MPa versus 8.34 ± 1.86 MPa for PC‐BU (*n* = 5) and PC‐BU‐SDF1α (*n* = 3), respectively, at 3 months. This estimated modulus was not significantly different from the explanted native carotids (*n* = 12, 6.64 ± 1.30 MPa) (p > 0.98) (Figure 6B). ePTFE grafts (*n* = 4) with an estimated modulus of 27.65 ± 7.54 MPa were significantly (*p* < 0.01) stiffer compared to TE grafts and the native carotid and did not alter during implantation.

### 2.7 Patency, Complications, and Function/Cannulation

**Figure 7:**
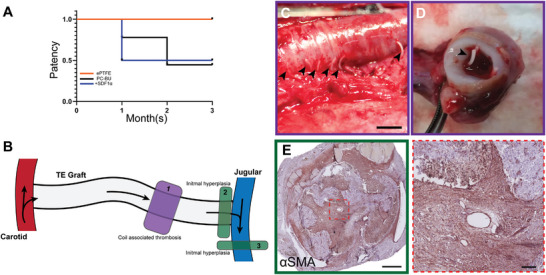
A) Patency and complications. Patency rates at the 1‐ and 3‐month timepoint, assessed with ultrasound, of PC‐BU, PC‐BU^+^SDF1α peptide, and ePTFE grafts. B) Location of stenosis complications in the graft, 1) associated with the coil, 2) intimal hyperplasia at the venous anastomoses, and 3) outflow vein. Outward fragmented ring observed at C) explantation and D) fragmentation inside of the lumen at 3 months. E) Immunohistochemistry on αSMA (Novared) in representative transverse sections at the venous anastomoses of the grafts explanted at 3 months show hyperplasia of myofibroblast cause stenosis. Scale bars 5 mm (C), 500 µm (E), and 50 µm (zoomed).

**Figure 8:**
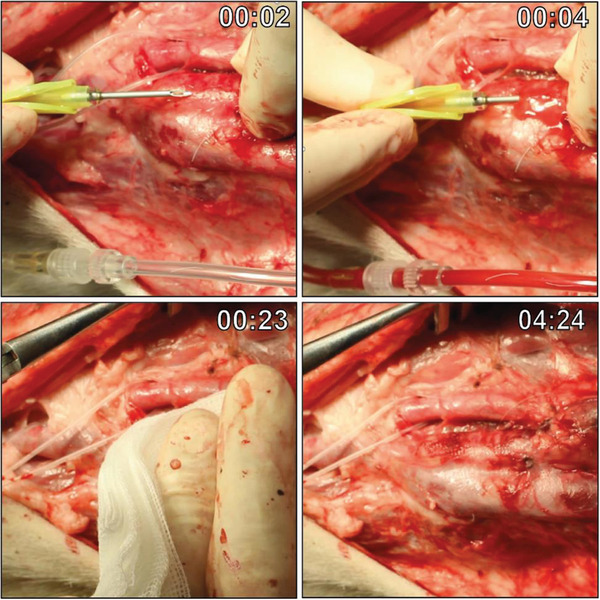
Cannulation of the graft. Timestamped images of cannulation with a 15 G dialysis needle dissected graft in an anaesthetized goat prior to explantation at 3 months. Hemostasis was achieved after a maximum of 5 min with slightly pressured gauze.

Patency (**Figure** **7**A) of our TE grafts checked monthly by ultrasound, showed that 70% of our TE grafts were patent versus 100% of the ePTFE grafts at 1 month. At 3 month explantation 50% of our TE grafts were fully patent versus 100% of the ePTFE graft due to occlusion, predominantly caused by stenosis at the venous anastomosis (*n* = 6/7) (Figure 7B). The reinforcing spiral was found fragmented during explanation at 3 months in a subset of grafts and fragments were incidentally found inside the lumen (Figure 7C) together with thrombus deposition. The majority of cells in the stenotic lesions near the venous anastomosis were αSMA‒positive fibroblasts (Figure 7D). No stenosis was observed in the first half of the graft near the arterial anastomosis. Grafts were successfully cannulated at termination (3 months) with a 15 G dialysis needle in fully anesthetized goats (**Figure** **8**, Video S2, Supporting Information). Hemostasis was acquired after a maximum of 5 min with slight gauze pressure.

## 3 Discussion

Our study shows that a fully synthetic biodegradable AV graft made of electrospun PC‐BU remodels in situ into autologous vascular living tissue in a large animal model. Over a 3 month period, we observed the simultaneous degradation of the synthetic electrospun PC‐BU material, cell infiltration, ECM formation, and neo‐vascular tissue formation, containing mature contractile markers and endothelial coverage. Importantly, the newly formed vessel was functional, compliant, and could be successfully cannulated with a 15 G dialysis needle, showing self‐healing capacity with time to hemostasis of a maximum of 5 min.

In situ vascular TE critically depends on balancing the breakdown of the scaffold material and neo‐tissue formation. Although we observed rapid resorption of the synthetic scaffold in our study compared to other scaffold materials,^[^ ^31^, ^32^, ^33^, ^34^, ^35^, ^36^, ^37^ ^]^ this did not lead to deformations, aneurysm formation, or ruptures, as has previously been reported.^[^ ^38^ ^]^ Within 3 months the synthetic scaffold was gradually replaced by autologous neo‐tissue over the whole length of the graft. The prolonged presence of scaffold material is associated with inflammation, fibrosis, and/or calcifications.^[^ ^19^, ^20^ ^]^ In our study, with a relatively limited presence of scaffold material, we did not observe calcification, fibrosis, abnormal cell proliferation and/or chronic inflammation in any of the grafts.

The inflammatory response, and specifically the phenotype of the infiltrating macrophages, plays an important role in the initiation and formation of neo‐tissue in the early stage of remodeling.^[^ ^39^ ^]^ We observed a high level of CD45^+^ leukocytes, M1 macrophage (iNOS^+^), and M2 macrophages (CD163^+^) in the explanted vascular grafts at 3 months, indicating a clear involvement of the immune reaction in the neo‐tissue formation. The relative abundance of macrophages of the immunomodulatory and tissue remodeling (M2) phenotype over the proinflammatory M1 phenotype, underscores the constructive remodeling response. The remodeling graft structure initially consisted of αSMA positive tissue at 1 month, which evolved into more mature contractile tissue indicated by the presence of calponin and myosin at 3 months with overall an absence of immune cells. Consistently, expression of Collagen I, a collagen type associated with later stages of neo‐tissue formation, increases over 3 months toward native levels. Interestingly, even without any pre‐seeding of the graft, we observed endothelial lining and capillary ingrowth already at 1 month after graft implantation.

The remodeling of our graft leads to fully autologous neo‐tissue with accommodating mechanical properties to the native vasculature, similar to autologous AV fistulas that have been shown to be associated with fewer long‐term vascular access events such as thrombosis, loss of primary patency, and interventions as compared to conventional synthetic grafts.^[^ ^5^, ^40^ ^]^ In our study, the constructive remodeling of the graft was not associated with improved patency compared to the unresponsive ePTFE grafts. PCL is known for its rigidity, which makes it convenient as an anti‐kinking solution, but has the possibility to fragment under repeated stress.^[^ ^41^ ^]^ In some cases, we observed thrombosis associated with fractured reinforcing spiral intruding into the lumen. This suggests the need for a more synchronized breakdown of tube and spiral materials in future applications. Additionally, we found cases of stenosis at the venous anastomosis, in the graft and native vasculature, similar to currently used vascular access grafts, due to a hypertrophic response leading to intimal hyperplasia (IH).^[^ ^42^, ^43^ ^]^ Of note, in our study no anticoagulation therapy was provided post‐implantation and no surgical or endovascular interventions were performed to maintain the functionality of the graft.

Previously, it has been shown that biofunctionalization of supramolecular polymer materials using short SDF1α peptides attracted and stimulated specific leukocyte populations in small diameter grafts in a rat model, suggesting that this biofunctionalization may accelerate subsequent neo‐tissue formation which could benefit graft patency.^[^ ^29^ ^]^ In our experiments, we observed no differences in cell infiltration, ECM deposition, vascular tissue formation, or graft patency between grafts with or without SDF1 biofunctionalization. This could implicate that other processes, like hemodynamic loading, proliferation, or ECM production dwarf the effect of specific immune cell attractants, such as SFD1α. This is supported by earlier work in in situ TE heart valves,^[^ ^44^ ^]^ where hemodynamic loading was shown to dominate tissue remodeling over scaffold properties. Alternatively, the rapid shift from synthetic fibers to neo‐tissue could have contributed to the rapid elimination of the bound peptide.

Compared to most studies of in situ vascular engineering, an important benefit of our study is the use of the clinically relevant large animal model.^[^ ^16^ ^]^ We limited anti‐coagulation to the implantation procedure, and none was given until explantation^[^ ^45^ ^]^ The goat model allowed us to use a graft with human dimensions and similar hemodynamic conditions.^[^ ^46^ ^]^ The animals’ long neck enabled an accessible implantation site, with the option for a straight configured graft that can be easily cannulated and imaged by ultrasound. The neo‐tissue formation in goats is similar to humans, in contrast to the similar‐sized porcine model, which is known to exhibit high re‐endothelialization capacity and growth at a more extensive rate compared to humans.^[^ ^47^, ^48^ ^]^

Our study revealed important issues that need further study to improve our vascular TE graft toward clinical translation. We encountered complications similar to current clinical practice, such as IH in the outflow vein. Although this confirms the clinical translatability of our goat model, it also illustrates the limitations of our approach, as in situ vascular TE can currently not solve the IH due to hemodynamic factors. Furthermore, our study used healthy goats, whereas the uremic environment present in dialysis patients may influence cell recruitment, scaffold degradation, and neo‐tissue formation.

## 4 Conclusion

In conclusion, in this study, we provide proof‐of‐principle that a synthetic, cell‐free, biodegradable, electrospun, anti‐kinking scaffold in a large animal AV shunt model can transform into an autologous living vascular graft within three months. The grafts are compliant and display a clear remodeling response and self‐healing capacity after cannulation. In our model of rapid scaffold breakdown and neo‐tissue replacement, SDF1α peptide biofunctionalization did not significantly influence vascular tissue formation or patency. Although the patency of our TE graft at 3 months was lower as compared to ePTFE, the transformation into autologous and compliant living tissue with self‐healing capacity may have long‐term advantages. Our modular supramolecular toolbox may allow for adjustment of graft and coil material to improve patency and be tailored to the specific needs of the individual patient.

## 5 Experimental Section

### Experimental Animals and Study Design

Twelve adult female Dutch milk goats, aged 1.5–2 years and weighing ≈65 kg were housed under standard climate‐controlled conditions in groups of up to 6, with ad libitum access to water and food. Animals were acclimatized for 7 days prior to the start of the study. The study protocol was approved by the Animal Ethics Committee of the University of Utrecht (CCD—AVD1150020173344). Four bilateral implantation groups were created (1: Bare scaffold vs Sham—2: Bare scaffold vs ePTFE—3: SDF1α peptide functionalized scaffold vs ePTFE—4: Bare scaffold vs SDF1α peptide functionalized scaffold) and animals were randomly divided between implantation groups. Procedures were performed bilaterally. On each side, an AV‐fistula was created between the internal jugular vein (IJV) and common carotid artery (CCA) using a bare supramolecular polymer graft (*n* = 9) made of polycarbonate‐bisurea (PC‐BU), an SDF1α peptide functionalized graft (*n* = 6), an ePTFE (GORE ACUSEAL *n* = 6) graft, and/or sham procedure was performed (*n* = 3) (Figure 1H). The patency of the grafts was monitored monthly using ultrasonography (CX50 ultrasound machine; Philips) (Figure 2E and Figure S2, Supporting Information). One (*n* = 2) and three months (*n* = 10) after implantation animals were euthanized and the grafts and proximate native vasculature were explanted for subsequent analyses. Sham operated jugular and carotid were explanted as controls for possible clamping damage. At termination, an angiogram was performed using a Mobile C‐arm (Philips PULSERA) (Figure 2D).

### Synthesis

PC‐BU (M_n_ = 21 kg mol^−1^) polymer ^[^ ^25^, ^26^ ^]^ (Figure 1A) and the bisurea‐SDF1α peptide^[^ ^28^ ^]^ (Figure 1A) were synthesized following the previously described procedures.

### Scaffold Fabrication

Grafts (8 cm length, 6 mm lumen diameter) were created by integrating a 3D printed PCL (M_n_ = 45 kg mol^−1^) spiral between two layers of electrospun PC‐BU scaffolds. Electrospun scaffolds were fabricated by dissolving PC‐BU polymer at a concentration of 23% w/v with a solvent mixture of 85% chloroform and 15% HFIP and electrospun using an IME Technologies electrospinning device. The first layer was electrospun with a solution flow rate of 70 µL min^−1^ while charged to 17 kV onto a 6 mm diameter stainless steel target in a climate‐controlled chamber maintained at 23 °C and 55% humidity. The nozzle was surrounded with chloroform vapor using a controlled gas shield module with a chloroform flow rate of 60 µL min^−1^ to reduce solvent evaporation upon extrusion. The nozzle was translated from the chuck securing the target 90 mm along the length of the target at a speed of 10 mm, with a turn delay of 600 ms at both ends. The target was rotated at a speed of 250 rpm and charged from 0 to −3 kV over the course of 900 s from the activation of the high voltage. The first layer was spun for 16 min ± 30 s with the thickness of the scaffold measured with a laser module while electrospinning, achieving a final thickness of 450 ± 50 µm. Next, the 3D printed spiral reinforcement was printed with an interspacing of 2.75 ± 0.25 mm at 60 °C using an Allevi 2 3D bioprinter directly onto the first electrospun later. The second layer of the polymer was spun immediately after 3D printing with the same parameters as the first layer, but with a spinning time of 7 min ± 30 s. At each end 1 cm was left without a spiral to enable cutting the correct angle during implantation. Grafts were then placed in a vacuum overnight to evaporate any remaining solvent then removed from the target and stored in a 50 mL falcon tube for transport. Prior to surgical implantation, grafts were sterilized using ethylene oxide by Synergy Health, Venlo in accordance with the certified standards ISO 11135 and ISO 13485. Fiber diameter was measured on SEM images with ImageJ with an average of 20 fibers per image.

### Surgical Procedure

Each animal received a buprenorphine skin patch (5 mg–0.005 mg h^−1^) two days prior to surgery. Prophylactic antibiotics consisting of Amoxicillin with Clavulanic acid was administered prior to the procedure (10 mg kg^−1^ I.V.). Animals were sedated with Medetomidine (0.04 mg kg^−1^ I.M.), initiated with and anesthetized with propofol (2 mg kg^−1^ I.V. bolus, followed by 10 mg kg^−1^ h^−1^ I.V. continuous infusion) and remifentanil (0.03 mg kg^−1^ h^−1^ I.V.). A midline incision in the neck was performed and the IJV and CCA were dissected bilaterally. Heparin (5000 IU I.V.) was administered 3 min before clamping the IJV. Grafts were cut at a 45° angle at both ends and were anastomosed in an end‐to‐side fashion from the ipsilateral CCA distally to the IJV proximally using running 7‐0 Prolene sutures (Ethicon #8683H) to create an AV conduit (Figure 2B). For sham procedures (always combined with a graft on the contralateral side) the vessels were only clamped, the duration of clamping was similar to the time used for the creation of the contralateral AV fistula. After removing the vascular clamps, vascular flow was confirmed in the jugular vein by a palpable thrill. In case of blood oozing through the graft wall light gauze pressure or absorbable surgicel (Ethicon #1903) was applied. The midline incision was closed using continuous 3.0 Vicryl subcutaneous sutures (Ethicon #V305H) and intracutaneous 3.0 Monocryl (Ethicon #W3650). For postoperative analgesia, a buprenorphine skin patch (5 mg–0.005 mg h^−1^) was given in combination with Meloxicam (0.5 mg kg^−1^ per day). Antibiotics, i.e., penicillin + dihydrostreptomycin (10 mg kg^−1^ I.M. per day) was given for 3 days.

### Sample Preparation and Histology

For histology and immunohistochemistry, tissues were fixed in 4% formaldehyde, processed, and embedded in paraffin. Sections (3 µm thickness) were stained with Hematoxylin and Eosin, Masson's trichrome, and picro sirius red (SR) using standard techniques. Sections were stained for elastin (SAB4200718, 1:1000; Sigma), eNOS (610296, 1:200, BD Transduction Laboratories), von Willebrand‐factor (ab6994, 1:400, Abcam), αSMA (ab7817,1:400, Abcam), Calponin (bs‐0095R,1:400, BIOSS), Myosin heavy chain (14‐6400‐82, Thermo Fisher Scientific), KI67 (RM‐9106, Thermo Scientific), CD45 (ab10558, 1:1000, Abcam), iNOS (ab15323, 1:400, Abcam), and CD163 (ab182422, 1:1000, Abcam). Whole sections were scanned and digitized with a NanoZoomer S360 (Hamamatsu C13220) or, for fluorescence, whole sections were imaged with a Leica DMi8 THUNDER Imager and analyzed by cell profiler.^[^ ^49^ ^]^

### Mechanical Analysis

From each explant, a 2–3 mm‐long ring section was cut from the vessel for uniaxial tensile ring testing. Immediately following sectioning, testing samples were gradually frozen in freezing medium (10% FCS, 10% DMSO in DMEM) in a Mr. Frosty and stored at −80 °C. Upon thawing, all samples were tested in one run and their mechanical properties were determined by a uniaxial tensile ring test (BioTester, 5 or 23 N load cell; CellScale) in combination with LabJoy software (V10.77, CellScale). The length, thickness, and inner diameter of the sections were measured with a calibrated microscope (VH‐Z20R lens, VHX‐500F; Keyence) and then placed onto custom‐made hooks of 1 mm in width to stretch the ring.^[^ ^50^ ^]^ The test started when a preload of 100 mN was reached. Each ring was preconditioned to 50% strain followed by a stretch to failure cycle, all at a strain rate of 100% per minute. The starting point of the analysis was set to the moment when the ring was only stretched around the hooks, excluding bending artifacts (Video S1, Supporting Information).

### RNA Isolation, cDNA Synthesis, and qPCR

Samples for RNA analyses were snap frozen in liquid nitrogen and stored at −80 °C. Total RNA was extracted from the samples using TRIzol reagent (Invitrogen). RNA (200 ng) was reverse transcribed using the Bioline SensiFAST cDNA Synthesis Kit (Bioline #BIO‐65054) and used for quantitative analysis of goat genes (Table S1, Supporting Information) with FastStart Universal SYBR Green (Roche #4913914001). B2M and 18S were used as standard housekeeping genes. The relative mRNA expression levels were determined using the ΔCT method.

### Cannulation

Grafts were cannulated in fully anesthetized animals preceding explantation with a 15 Gauge dialysis needle (Hospal #A15L15SGP+) attached to a free‐flowing line. After cannulation, slight pressure was applied for a maximum of 5 min till hemostasis was achieved.

### Statistics

Prior to commencing the animal experiments, a power calculation (α:0.05, β:0.90) was performed with G*power, *t*‐test with two groups one‐way, based on cell infiltration data in similar in vivo experiments in the group. Data are presented as means ± standard error of the mean (SEM) and considered statistically significant for *p* <  0.05. *T*‐tests and 1‐way ANOVA with repeated measures (Tukey posthoc) were used as appropriate, using GraphPad Prism 9 software (GraphPad Software).

### Ethics Approval and Consent to Participate

The study protocol was approved by the Animal Ethics Committee of the University of Utrecht (CCD—AVD1150020173344).

## Conflict of Interest

The authors declare no conflict of interest.

## Author Contributions

P.J.B., W.S., M.T., R.J.T., D.J.W., J.O.F., C.V.C.B., P.Y.W.D., G.J.B., and M.C.V.: Conception and design of the work; P.J.B., W.O., R.C.H.D., K.d.O., H.M.J., M.v.d.K., P.M.d.B.: Data collection; P.J.B., W.O., J.O.F., R.C.H.D., P.M.d.B., M.C.V.: Data analysis and interpretation; P.J.B., J.O.F., M.T., M.C.V.: Drafting the article; P.J.B., J.O.F., M.T., R.J.T., D.J.W., R.C.H.D., C.V.C.B., P.Y.W.D., G.J.B., M.C.V.: Critical revision of the article.

## Supporting information

Supporting Information

Supplemental Video 1

Supplemental Video 2

## Data Availability

The data that support the findings of this study are available from the corresponding author upon reasonable request.
