# Supplementary material for: Off‐the‐Shelf Synthetic Biodegradable Grafts Transform In Situ into a Living Arteriovenous Fistula in a Large Animal Model
Source: Adv Healthc Mater. 2024 Mar 26;13(17):2303888. doi: 10.1002/adhm.202303888 (PMC11469054; doi:10.1002/adhm.202303888)
Supplement: Supplementary file 1 — Supporting Information [file ADHM-13-2303888-s001.pdf]

ADVANCED HEALTHCARE MATERIALS

Supporting Information

for *Adv. Healthcare Mater.*, DOI 10.1002/adhm.202303888

Off-the-Shelf Synthetic Biodegradable Grafts Transform In Situ into a Living Arteriovenous Fistula in a Large Animal Model

*Paul J. Besseling, Wojciech Szymczyk, Martin Teraa, Raechel J. Toorop, Dan Jing Wu, Rob C. H. Driessen, Arturo M. Lichauco, Henk M. Janssen, Melanie van de Kaa, Krista den Ouden, Petra M. de Bree, Joost O. Fledderus, Carlijn V. C. Bouten, Gert J. de Borst, Patricia Y. W. Dankers and Marianne C. Verhaar**

Supplemental tables and figures

Table S1: qPCR primer list

Target		*Sequence (5'--> 3')*	*Tm (°C)*	*Ampl. Size*	*NCBI reference*
**B2M**	F	TGTCCCACGCTGAGTTCACT	60.00	137	XM_012180604.1
	R	TGAGGCATCGTCAGACCTTGA			
**Collagen I**	F	CCCTTCTTGGTCAGACTCCC	61,5	166	XM_018055997.1
	R	GGCTGGCTAGAGGGGATAAA			
**Collagen III**	F	AACGGCATCAAAGGACATCG	62	158	XM_005675869.3
	R	CCACTTGTTCCATCCTTGCC			
**18S**	F	ATGCAGAATCCACGCCAATAC	58.00	147	DQ149973.1
	R	GGCCCGAATCTTCTTCAGG			

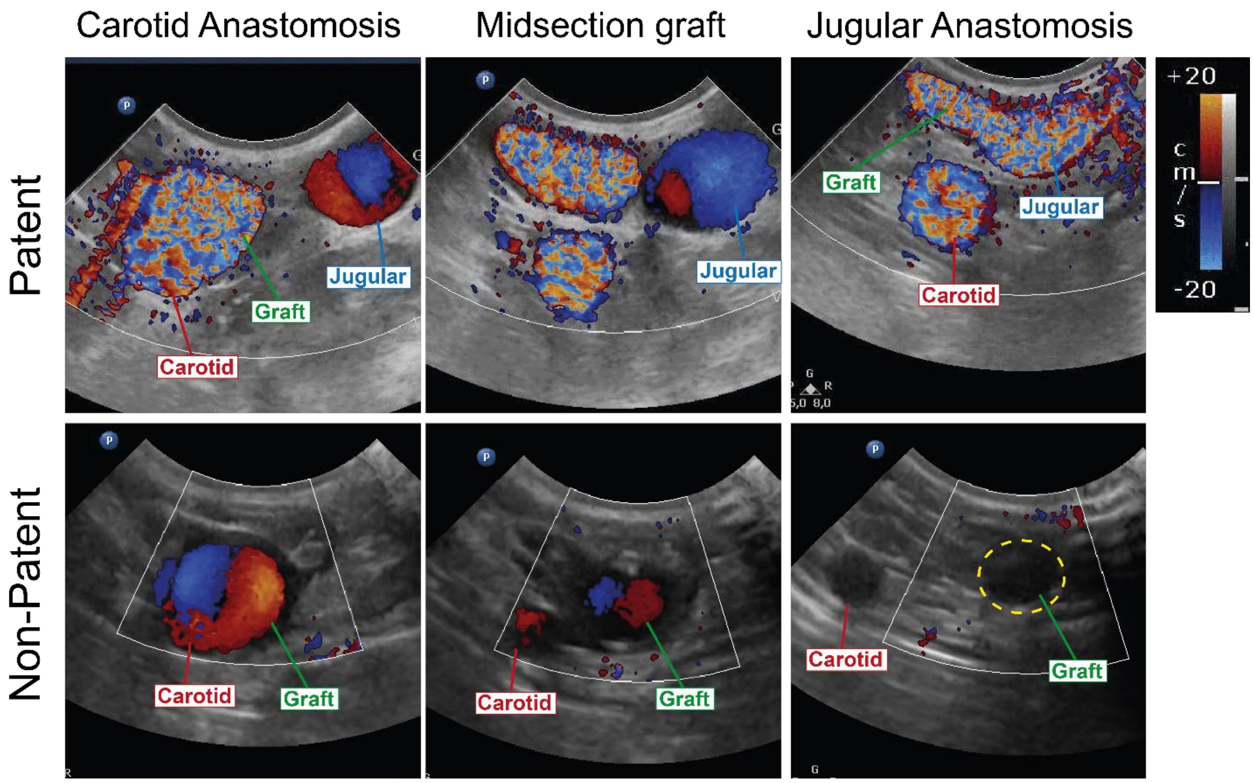

Figure S1 – Color Doppler images of patent and non-patent grafts. Transverse images of the carotid anastomosis, midsection of the graft and jugular anastomosis. Non-patent graft indicated with yellow dashed line. Bidirectional flow indicated by separate blue and red flow patterns, as opposed to irregular flow pattern in patent graft and outflow vein.

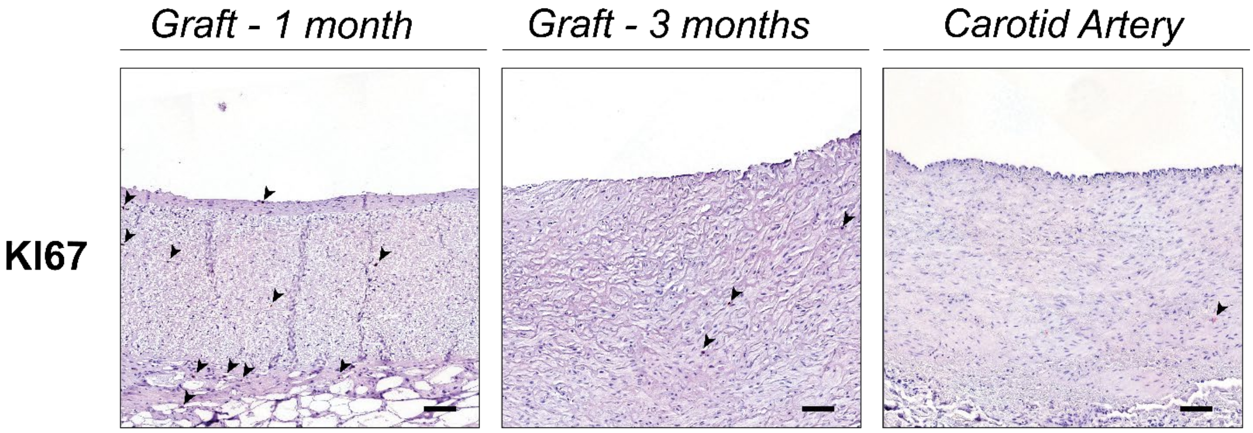

Figure S2 – Immunohistochemistry of proliferation marker KI67. Transverse sections (centre part of the grafts), explanted at 1 and 3 months and the native carotid artery as control. Stained with KI67 (Novared®). Black arrows indicate positive cells. Scale bars 100μm

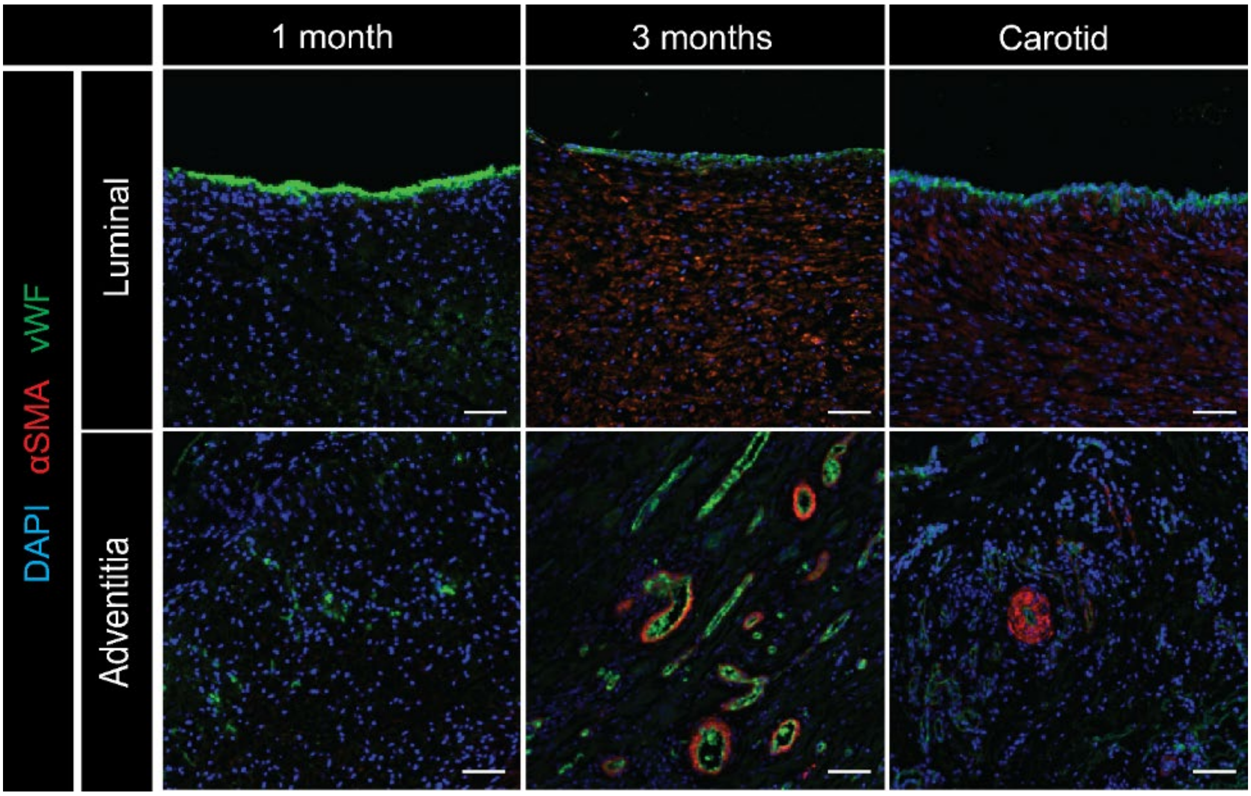

Figure S3 – Immunohistochemistry of endothelial lining and capillary ingrowth. Luminal and adventitial images of transverse sections (centre part of the grafts), explanted at 1 and 3 months and the native carotid artery as control. Stained with DAPI, smooth muscle marker αSMA (red), endothelial marker von Willebrand factor (green). Scale bars 50µm.
